# Emicizumab prophylaxis for people with hemophilia A: Waste estimation and the Brazilian perspective

**DOI:** 10.1016/j.jsps.2023.101867

**Published:** 2023-11-10

**Authors:** Ricardo Mesquita Camelo, Mariana Michel Barbosa, Luila Clicia Moura Henriques, Antony Paul Martin, Brian Godman, Augusto Afonso Guerra Júnior, Francisco de Assis Acurcio, Juliana Alvares-Teodoro

**Affiliations:** a Faculty of Pharmacy, Universidade Federal de Minas Gerais, Belo Horizonte, Brazil; b QC Medica, Liverpool, United Kingdom; c Faculty of Health and Life Science, University of Liverpool, Liverpool, United Kindgom; d Department of Public Health Pharmacy and Management, School of Pharmacy, Sefako Makgatho Health Sciences University, Ga-Rankuwa, South Africa; e Centre of Medical and Bio-Allied Health Sciences Research, Ajman University, Ajman, United Arab Emirates; f Strathclyde Institute of Pharmacy and Biomedical Science, University of Strathclyde, Glasgow, United Kingdom

**Keywords:** hemophilia A, emicizumab, prophylaxis, waste, budget impact

## Abstract

Costs of hemophilia A treatment are increasing. Waste of clotting products should be avoided. To estimate the first-year waste of emicizumab prophylaxis for people with hemophilia A and inhibitors (PwHAi) who failed immune tolerance induction (ITI), in Brazil. We evaluated the manufacturer and the Brazilian Ministry of Health (MoH) protocol-recommended regimens in a budget impact model. The loading dose consisted of 3.0 mg/kg/Q1W for 4 weeks, for both recommendations. The manufacturer maintenance regimens comprised 1.5 mg/kg/Q1W, 3.0 mg/kg/Q2W, and 6.0 mg/kg/Q4W. The MoH protocol maintenance regimen encompassed a hybrid Q1W/Q2W administration, depending on the body weight. The Q4W regimen was not recommended by the MoH protocol. Analyses were performed to estimate waste given its expense based on the World Health Organization body weight range (percentiles [P] 15, 50, and 85). The first-year emicizumab waste was estimated individually and for the disclosed PwHAi who failed ITI (n = 114). The highest emicizumab waste was estimated for the lowest body weights and the Q1W regimen. The Q4W regimen resulted in the lowest emicizumab waste, followed by the MoH protocol regimen. The total reconstituted costs estimated for the PwHAi who failed ITI according to the hybrid MoH protocol ranged from US$32,858,777 (P15) to US$47,186,858 (P85), with emicizumab waste ranging from 7.9 % (US$2,594,515) to 3.7 % (US$1,738,750), respectively. Lost resources due to current protocols for emicizumab prophylaxis for PwHAi who failed ITI in Brazil are considerable. Waste was more pronounced due to lower body weight and shorter administration intervals.

## 1 Introduction

In recent years, treatment costs of several diseases have increased, including rare diseases (Rome et al., 2022, Vincent Rajkumar, 2020). Suggested reasons for this tendency are the monopoly of companies in producing and providing medicines, the severity of the disease, and the high costs of developing new technologies (Godman et al., 2021, Luzzatto et al., 2018, Vincent Rajkumar, 2020). However, higher threshold levels for new medicines for one specific disease need to be balanced against the needs of patients with other diseases in countries with universal healthcare systems and finite resources. Consequently, it is imperative that any resources to treat rare diseases are used as wisely as possible.

This includes hemophilia A, which is a rare inherited bleeding disorder due to a deficiency of the clotting activity of factor VIII (FVIII) (Berntorp et al., 2021). As a consequence, traumatic and spontaneous bleeds may occur, leading to health complications including arthropathy, reduced health-related quality of life (HRQoL), and increased risk of death (Hay et al., 2021, Tagliaferri et al., 2015). Consequently, for those people with hemophilia A (PwHA) and a bleeding phenotype, the regular replacement of FVIII to avoid hemorrhages (i.e., prophylaxis) is recommended as the standard of care (Srivastava et al., 2020). Prophylaxis hinders the worsening of HRQoL and reduces the hemorrhage-related mortality of PwHA (Hay et al., 2021, Tagliaferri et al., 2015). However, the development of neutralizing anti-FVIII antibodies (inhibitors) in up to 30 % of PwHA renders FVIII replacement ineffective (Schep et al., 2018). For PwHA and inhibitors (PwHAi), prophylaxis with bypassing agents offers an alternative treatment option (Meeks and Batsuli, 2016). Since both FVIII and bypassing agents account for more than 90 % of the direct medical care costs of hemophilia A (Café et al., 2019, Cortesi et al., 2018), the price and the consumed amount of these concentrates are the main determinants of the treatment cost.

New technologies, including non-factor products and gene therapy, are being introduced as alternative treatments for hemophilia A (Nogami and Shima, 2023). However, as many of these treatments are associated with high costs, it is imperative to assure their quality, effectiveness, and safety (Rodriguez-Merchan, 2020). In addition, it is imperative to avoid product waste, which may assume proportions of almost half of the expenditures of the treatment (Ghosh and Ghosh, 2021, Hackbarth, 2012).

Emicizumab is a bispecific humanized antibody that mimics the FVIII clotting activity (Kitazawa and Shima, 2020). Since 2018, emicizumab has been indicated as prophylaxis for PwHA without and with inhibitors based on single doses every week (Q1W), every 2 weeks (Q2W), or every 4 weeks (Q4W) (Srivastava et al., 2020). In addition, safety issues that were described in the pivotal studies are rare in the real-world, after therapy adjustments were implemented (Makris et al., 2019, Oldenburg et al., 2017). Since the current posology is based on body weight, the discard of remaining treatment in the vial is frequent. In a real-world setting, mean emicizumab waste reached 8.4 % of the reconstituted vials (Sun et al., 2022). In comparison, the waste of FVIII was negligible (Sun et al., 2022). Considering the high expenditures for prophylaxis with emicizumab that may reach €109,712,238 (US$123,315,699),^a^ for children, and €253,240,465 (US$284,616,959), for adolescents/adults, in the first 5 years of use (Mancuso et al., 2022), any waste translates a misuse of financial resources.

The impact of the total cost of the treatment and the consequences of emicizumab waste may be even more significant in developing countries where available resources to treat people with rare diseases are a greater concern (Ghosh and Ghosh, 2021). Currently, Brazil has the fourth largest population of PwHA in the world (World Federation of Haemophilia, 2022). Emicizumab was incorporated into the public Unified Healthcare System (Sistema Único de Saúde, SUS) in 2019 as prophylaxis for PwHAi who failed immune tolerance induction (ITI) (Brasil, 2021, Brasil, 2019). In this context, we aimed to estimate the potential waste of emicizumab and associated costs during the first year of use in Brazil comparing the regimens recommended by the Ministry of Health (MoH), in comparison to regimens recommended by the manufacturer. Finally, we discussed possible options to reduce waste and associated costs.

## 2 Methods

### 2.1 Study design and data sources

We performed a budget impact analysis of the first year of emicizumab use as prophylaxis against bleeding events for male PwHAi who failed ITI. We based our analysis on the following data sources:

1. Number of PwHAi who failed ITI who were eligible for initial use of emicizumab in Brazil: National Committee for Health Technology Incorporation (CONITEC) Recommendation Report for emicizumab (p.51) (Brasil, 2019);
2. Number of PwHAi who failed ITI per age group in Brazil: BrazIT Study (Camelo et al., 2022);
3. Male body weight percentiles according to age: WHO datasheet (Table sT1) (“https://www.who.int/tools,” n.d.);
4. Emicizumab regimens: manufacturer recommendation leaflet and CONITEC Protocol for Emicizumab Use (MoH protocol) (Brasil, 2021, https://www.hemlibra-hcp.com/dosing-and-administration/dosing-calculator.html, n.d.);
5. Vial reconstitution according to the prescription dose: manufacturer emicizumab dose calculator and the MoH protocol (Brasil, 2021, https://www.hemlibra-hcp.com/dosing-and-administration/dosing-calculator.html, n.d.).

WHO weights, estimated number of PwHAi who failed ITI, and the final calculations of costs were rounded to reach entire numbers. When the last decimals were ≥ 5, the number was rounded up; when the last decimals were < 5, the number was rounded down. Negative values reported as underdosage (amounts) or savings (costs). When negative-valued parameter was used in calculations, it was determined as 0 (zero).

Due to the study design, ethical approval was not required.

### 2.2 Emicizumab regimens and vial selection

Available emicizumab vials contained 30 mg, 60 mg, 105 mg, and 150 mg (Fig. s1). The loading regimen of emicizumab was the same for both the manufacturer recommendation leaflet and the MoH protocol (Brasil, 2021, https://www.hemlibra-hcp.com/dosing-and-administration/dosing-calculator.html, n.d.): 3.0 mg/kg Q1W for 4 weeks. The maintenance regimens recommended by the manufacturer were 1.5 mg/kg Q1W, 3.0 mg/kg Q2W, and 6.0 mg/kg Q4W. There were no criteria to recommend one regimen over another. Body weight value and unit were inputted in a web-based calculator sponsored by the manufacturer, designed to help the prescriber select the best option of available vials to avoid emicizumab waste.

The MoH protocol did not recommend the Q4W-maintenace regimen (Brasil, 2021). Otherwise, a hybrid protocol alternating Q1W- and Q2W-maintenance regimens was recommended, based on body weight (Table sT2). In addition, the best option of available vials was indicated for selection.

### 2.3 Waste estimation

We calculated the emicizumab waste for each recommendation described above using a two-step process: firstly, we calculated individual (per person) emicizumab waste according to the body weight; finally, we estimated the emicizumab waste for the total population recommended by the MoH protocol (Brasil, 2021). We assumed the individual would remain in the same recommended regimen and their body weight would not change during the treatment period. The total emicizumab required for the first year of use (52 weeks) was the sum of the loading and the maintenance doses. Emicizumab waste was the difference between the reconstituted (entire selected vial) and the prescribed (to be infused) emicizumab, corresponding to the leftover product. The amounts and costs were expressed as absolute (mg, for amounts, and Purchasing Power Parity [PPP] US$, for costs) or relative values (calculated as wasted per reconstituted emicizumab).

For the individual analyses, we estimated only the amounts of emicizumab (mg). Calculations were performed for each 1-kg body weight per individual, from 10 to 100 kg.

The MoH protocol recommended emicizumab only for PwHAi who failed ITI (Brasil, 2021). We estimated the number of eligible PwHAi from the CONITEC Recommendation Report for emicizumab (Brasil, 2019). This population was stratified into age groups: 1 year per group, from 2 to 18 years, and a cluster group of ≥ 19 years. We based our calculations on the age distribution of PwHAi who failed ITI described by the BrazIT Study to perform this stratification (Camelo et al., 2022). As a result, we obtained the estimated number of individuals per age group. For each age group, we estimated the body weight as a range. Body weight percentiles (P) 15, 50, and 85 were obtained from the WHO datasheet for males aged from 2 years to ≥ 19 years (“https://www.who.int/tools,” n.d.). For this group, the total costs of emicizumab therapy and emicizumab waste were calculated.

### 2.4 Emicizumab costs

The price of emicizumab (mg) purchased by the MoH was R$229.55 (“https://www.gov.br/saude/pt-br/acesso-a-informacao/licitacoes-e-contratos/contratos-dlog/dlog-2021/ct-141–2021-emicizumabe-de-30-mgml-e-150-mgml-injetavel-25000–100202-2020–33-10–06-2021-produtos-roche-quimicos-e-farmaceuticos-s-a.pdf/view,” n.d.). Using the PPP dollar for the year 2021 (US$1.00 = R$ 2.53) (OECD (2023)https://doi.org/10.1787/1290ee5a-en), the converted price of emicizumab was US$90.73/mg.

## 3 Results

### 3.1 Individual analyses

**Fig. 1:**
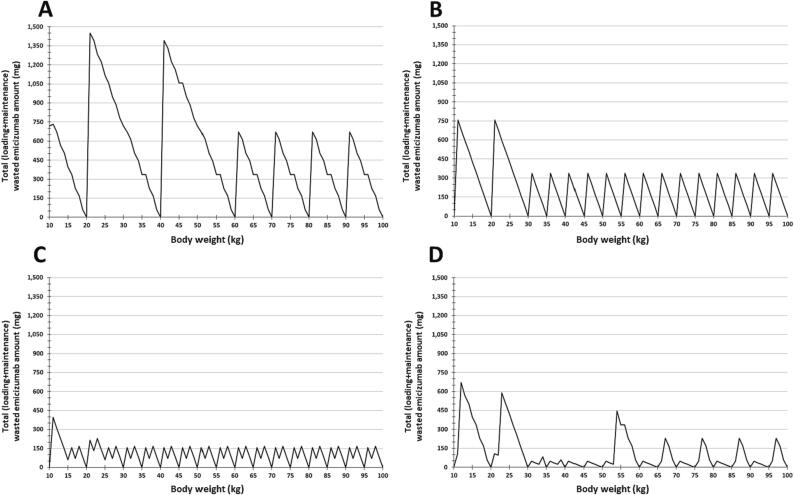
Absolute total wasted emicizumab after reconstitution per individual body weight, during the first year of treatment. The estimation included the loading doses and the maintenance regimens, according to the manufacturer leaflet (**A** to **C**) and the CONITEC Protocol of Emicizumab Use for the Brazilian Ministry of Health recommendations (**D**). (**A**) Maintenance doses every 1 week. (**B**) Maintenance doses every 2 weeks. (**C**) Maintenance doses every 4 weeks. (**D**) Maintenance doses at a hybrid of every 1-week and every 2-week administration. CONITEC, Comissão Nacional de Incorporação de Tecnologia (National Committee for Technology Incorporation).

**Fig. 2:**
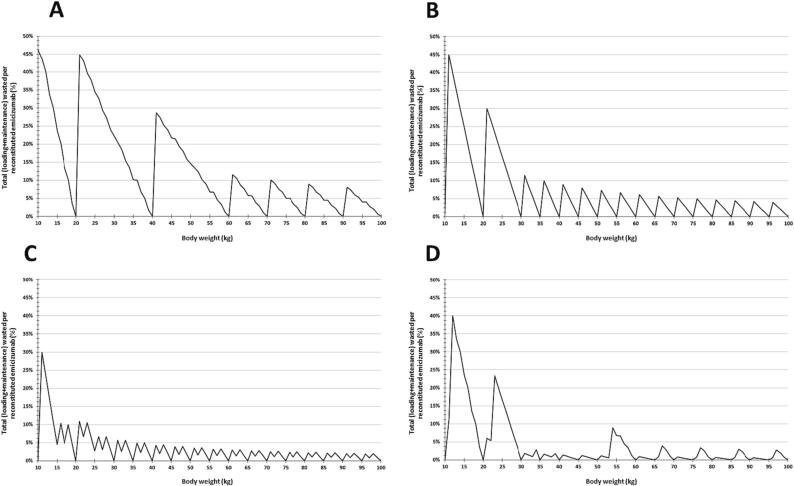
Percentage of wasted per reconstituted amount of emicizumab per individual body weight, during the first year of treatment. The estimation included the loading doses and the maintenance regimens, according to the manufacturer leaflet (**A** to **C**) and the CONITEC Protocol of Emicizumab Use for the Brazilian Ministry of Health recommendations (**D**). (**A**) Maintenance doses every 1 week. (**B**) Maintenance doses every 2 weeks. (**C**) Maintenance doses every 4 weeks. (**D**) Maintenance doses at a hybrid of every 1-week and every 2-week administration. CONITEC, Comissão Nacional de Incorporação de Tecnologia (National Committee for Technology Incorporation).

The results of the reconstituted, prescribed, and wasted emicizumab amounts for loading and maintenance regimens during the first year of use per individual 1-kg body weight are depicted in Tables sT3 and sT4. The total absolute amount of emicizumab wasted per individual 1-kg body weight is shown in Fig. 1. The total relative amount of wasted per reconstituted emicizumab per individual 1-kg body weight is shown in Fig. 2. The graphics depicting all the regimens together are shown in Fig. s2.

Among the manufacturer-recommended regimens, the Q1W and the Q4W regimens were associated with the highest and the lowest estimates of both total absolute and relative emicizumab waste, respectively. Higher emicizumab waste was found in the lowest body weights, but the range differed among the regimens: between 10 and 60 kg, for the Q1W regimen; between 10 and 30 kg, for the Q2W regimen; and between 10 and 20 kg, for the Q4W regimen. The highest total relative emicizumab waste was found in P15 for Q1W (46 %, 10 kg). After reaching these upper levels, the total amount of emicizumab waste reduced and remained constant. However, for the total relative emicizumab waste, the percentage remained reducing, albeit slowly. Zero emicizumab waste was found more frequently for the Q2W and the Q4W regimens (17 times; 19 %), which was considered a coincidental finding. There were seven (8 %) body weights for which the emicizumab waste was zero in the Q1W regimen. Finally, the manufacturer regimens did not result in underdosing.

For the MoH protocol-recommended regimen, total absolute and relative emicizumab waste varied considerably according to the individual 1-kg body weight. The lowest body weights (from 10 to 30 kg) were associated with the greatest amount of emicizumab waste. The highest total relative emicizumab (40 %) waste was found in the third lowest body weight. Zero emicizumab waste was found in 16 (18 %) body weights, and underdosage up to −9% of the manufacturer regimen recommendation occurred in 44 (48 %) body weights.

### 3.2 Brazilian estimative analyses

**Fig. 3:**
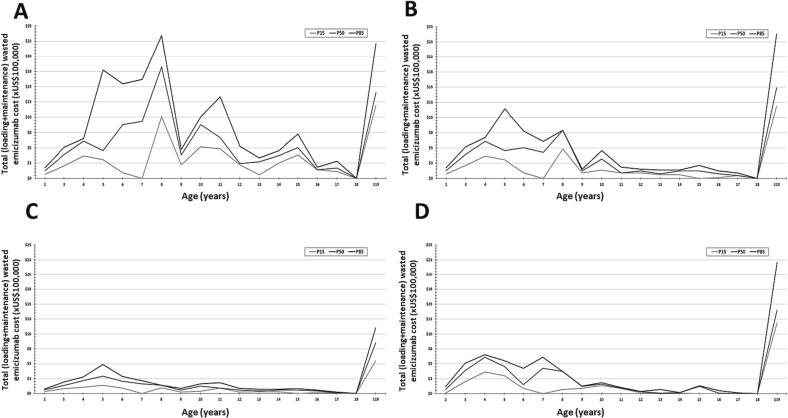
Cost of total wasted emicizumab during the first year of treatment per age, stratified by the body weight percentile, according to the estimated age distribution of people with hemophilia A and inhibitors who failed immune tolerance induction. The estimation included the loading doses and the maintenance regimens and the choice of the vials proposed by the manufacturer leaflet (**A** to **C**) or the CONITEC Protocol of Emicizumab Use for the Brazilian Ministry of Health (**D**). (**A**) Maintenance doses every 1 week. (**B**) Maintenance doses every 2 weeks. (**C**) Maintenance doses every 4 weeks. (**D**) Maintenance doses at a hybrid of every 1-week and every 2-week administration. CONITEC, Comissão Nacional de Incorporação de Tecnologia (National Committee for Technology Incorporation); P15, 15th percentile body weight; P50, 50th percentile body weight; P85, 85th percentile body weight.

The estimated number of Brazilian PwHAi who failed ITI per age range is depicted in Table sT5. The total cost of emicizumab waste per age group is shown in Fig. 3 and Table sT6. The age-stratified and total costs of emicizumab treatment and emicizumab waste during the first year for the 114 PwHAi who failed ITI (Brasil, 2019) whose body weight was estimated based on the results of the BrazIT Study (Camelo et al., 2022) are depicted in Table 1.

**Table 1:** Age-stratified and total costs of emicizumab treatment and emicizumab waste during the first year of treatment of the people with hemophilia A and inhibitors who failed immune tolerance induction in Brazil.

**AGE INTERVAL (YEARS)**	**ESTIMATED NUMBER OF PwHAi**	**EMICIZUMAB REGIMEN**	**P15**				**P50**				**P85**		
			**RECONSTITUTED (US$)**	**WASTED (US$)**	**RATIO (%)**		**RECONSTITUTED (US$)**	**WASTED (US$)**	**RATIO (%)**		**RECONSTITUTED (US$)**	**WASTED (US$)**	**RATIO (%)**
2 to 5	23	Q1W	3,505,807	941,777	26.9		3,505,807	640,191	18.3		4,921,195	1,528,619	31.1
		Q2W	3,505,807	952,665	27.2		3,505,807	640,191	18.3		4,267,939	914,558	21.4
		Q4W	2,917,877	364,735	12.5		3,211,842	346.226	10.8		3,680,009	326,628	8.9
		MoH	3,440,482	885,162	25.7		3,505,807	640,191	18.3		3,614,683	309,208	8.6
6 to 9	24	Q1W	5,073,622	1,323,932	26.1		7,055,165	2,693,592	38.2		7,066,052	1,968,478	27.9
		Q2W	4,420,366	670,676	15.2		5,487,350	1,173,683	21.4		5,563,564	487,764	8.8
		Q4W	3,963,086	213,397	5.4		4,670,780	357,113	7.6		5,236,936	161,136	3.1
		MoH	3,897,761	252,592	6.5		5,095,397	820,925	16.1		5,498,238	461,634	8.4
10 to 13	18	Q1W	5,334,924	1,282,559	24.0		5,694,215	781,730	13.7		7,174,928	1,134,488	15.8
		Q2W	4,420,366	381,066	8.6		5,106,284	228,640	4.5		6,325,696	320,095	5.1
		Q4W	4,224,389	185,089	4.4		5,008,296	130,651	2.6		6,195,044	189,444	3.1
		MoH	4,224,389	250,415	5.9		4,844,982	50,083	1.0		5,803,091	97,988	1.7
14 to 17	10	Q1W	4,442,141	888,428	20.0		4,844,982	305,009	6.3		6,053,506	455,102	7.5
		Q2W	3,658,234	121,941	3.3		4,649,005	243,882	5.2		5,792,203	198,154	3.4
		Q4W	3,592,908	56,616	1.6		4,518,354	113,231	2.5		5,661,552	67,503	1.2
		MoH	3,462,257	17,420	0.5		4,453,028	139,361	3.1		5,520,013	63,148	1.1
≥18	39	Q1W	17,833,889	1,188,926	6.7		20,806,204	212,308	1.0		26,750,833	806,771	3.0
		Q2W	17,833,889	1,188,926	6.7		20,806,204	297,231	1.4		26,750,833	891,694	3.3
		Q4W	17,196,964	552,001	3.2		20,806,204	297,231	1.4		26,113,909	254,770	1.0
		MoH	17,833,889	1,188,926	6.7		20,806,204	212,308	1.0		26,750,833	806,771	3.0
TOTAL	114	Q1W	36,190,382	5,625,623	15.5		41,906,372	4,632,830	11.1		51,966,515	5,893,458	11.3
		Q2W	33,838,661	3,315,274	9.8		39,554,651	2,583,627	6.5		48,700,235	2,812,267	5.8
		Q4W	31,895,224	1,371,838	4.3		38,215,476	1,244,453	3.3		46,887,449	999,482	2.1
		MoH	32,858,777	2,594,515	7.9		38,705,418	1,862,868	4.8		47,186,858	1,738,750	3.7

Ages were randomly stratified into 4-year groups.

MoH, Brazilian Ministry of Health; P15, 15th percentile body weight; P50, 50th percentile body weight; P85, 85th percentile body weight; Q1W, every 1 week; Q2W, every 2 weeks; Q4W, every 4 weeks; US$, United States dollar.

The ages ranged from 2 to 70 years. Overall and despite the body weight percentiles, both total emicizumab treatment and total emicizumab waste costs were higher for the Q1W followed by the Q2W maintenance regimens recommended by the manufacturer and the MoH protocol. The lowest costs were found for the Q4W maintenance regimen recommended by the manufacturer.

As an example, for the 50th percentile, the total costs of emicizumab treatment ranged from US$38,215,476 (Q4W) to US$41,906,372 (Q1W). Their respective total costs of emicizumab waste ranged from US$1,244,453 or 3.3 % of the total cost of emicizumab treatment to US$4,632,830 or 11.1 % of the total cost of emicizumab treatment. The total costs of emicizumab treatment and emicizumab waste according to the MoH protocol were US$38,705,418 and US$1,862,868 (4.8 % of the total cost of emicizumab treatment), respectively.

## 4 Discussion

The treatment of rare diseases with biological medicines is described as one of the costliest budget items in public health (Luzzatto et al., 2018, Rome et al., 2022, Vincent Rajkumar, 2020). For hemophilia A, emicizumab is currently recommended as an effective biotherapeutic for prophylaxis against bleeding events across countries (Callaghan et al., 2021, Srivastava et al., 2020). Since emicizumab is available in a few presentations and the regimens recommended by the manufacturer are rigid (Mahlangu et al., 2022, Srivastava et al., 2020), the product waste and associated costs may be significant. We estimated emicizumab waste and associated costs during the first year of prophylaxis according to the MoH protocol (Brasil, 2021, Brasil, 2019). The total expenditures varied between US$32,858,777 (P15) and US$47,186,858 (P85), with respective total relative emicizumab wastes varied between 7.9 % (US$2,594,515) and 3.7 % (US$1,738,750). The wastes resulting from the estimation of the MoH protocol regimen were lower than the wastes resulting from the Q1W and Q2W regimens, although still higher than the Q4W regimen.

Historically, waste of factor products was considered minimal, mainly representing expiry or premature discharge due to processing errors (Arnold et al., 2007, Sun et al., 2022). Rounding doses to the nearest vial size is a common practice to avoid waste and no safety issues have been reported to date (Kempton and White, 2009). However, waste has been recently introduced to hemophilia A economic analyses following the advent of emicizumab (Mancuso et al., 2022, Sun et al., 2022). Our estimations for total relative emicizumab wastes for a PwHA with a P50 body weight on Q1W and Q2W regimens were 11.1 % and 6.5 %, respectively, similar to previous findings (Sun et al., 2022). In addition, the waste extent inversely varied with body weight: emicizumab waste and associated costs were higher in the lowest body weight and lower in the highest body weights, also corroborating with previous reports (Mancuso et al., 2022). However, both studies did not evaluate the Q4W regimen recommended by the manufacturer (Mancuso et al., 2022, Sun et al., 2022). We found that emicizumab waste was lower for the Q4W regimen than for Q1W and Q2W regimens. Interestingly, the MoH protocol did not recommend the Q4W regimen either with no explanation for this decision (Brasil, 2021). The first publications of the effectiveness of the Q4W regimen dated 2019 (Pipe et al., 2019, Young et al., 2019), resulting in a short period of experience with this regimen until the MoH protocol was published in 2021 (Brasil, 2021). In addition, it remains unclear if the Q4W regimen was as effective as the Q1W and Q2W regimens in preventing bleeding events (Callaghan et al., 2021, Schmitt et al., 2023). A review of seven clinical studies described 24 (among 675) PwHA who needed emicizumab dose up-titration because of suboptimal bleeding prevention. The authors found that relatively more PwHA on the Q4W regimen (6.6 %) required dose up-titration than those PwHA prescribed the Q1W (2.8 %) and Q2W regimens (1.5 %) (Schmitt et al., 2023). Nevertheless, an analysis of the long-term results of the pilot trials of emicizumab prophylaxis for PwHAi showed that the Q1W and Q2W regimens recommended by the MoH protocol had similar efficacy in reducing the bleeding rates (Callaghan et al., 2021). In addition, the quality of life of PwHAi on Q1W, Q2W, and Q4W emicizumab regimens were similar (Mancuso et al., 2020).

Preventing drug waste and associated costs is important in developing countries such as Brazil (Ghosh and Ghosh, 2021). These savings may allow more PwHA to be treated with emicizumab. We should mention that the MoH recently expanded emicizumab prophylaxis to all PwHAi (Brasil, 2023). Different and associated strategies may be implemented to prevent emicizumab waste (Table 2). Regardless of the chosen approach, the establishment or the development of a guideline addressing such strategies based on scientific data on effectiveness and safety are essential (D’Albini et al., 2023, Fasola et al., 2014, Srivastava et al., 2020). This guideline may encompass different healthcare professionals since every person may take part in the waste prevention process (D’Albini et al., 2023). Compliance with the guideline should also be monitored and deviations should be solved on an individual basis (D’Albini et al., 2023, Yamada et al., 2020). Adopting validated management tools may also be a robust alternative (Damuzzo et al., 2019, Heitmiller et al., 2010). Finally, decision-makers may involve assistant healthcare professionals to collaborate in the process of health performance technology assessment (Berwick and Hackbarth, 2012, Lee et al., 2022). The next step has a two-sided approach: (a) continuing education of healthcare professionals about treatment cost and strategies to avoid drug waste and (b) frequent incentive of PwHA to comply with the prescribed treatment, including the best practice to guarantee hemostasis effectiveness without emicizumab waste (Bekker et al., 2018, Bewley and Page, 2011, Srivastava et al., 2020, Tisdall et al., 2019, West and Cordina, 2019). As an example, in Brazil, PwHA are treated at the SUS-based hemophilia treatment centers distributed throughout the country, where they receive integrated assistance and hemostatic products (Ferreira et al., 2014). The Pharmacist composing this interdisciplinary team has many roles, including the coordination to optimize drug therapy, involving the assurance of compliance to protocols and the avoidance of adverse events, and the patient education and promotion of treatment adherence (“Diário Oficial da União. Resolução no 673, de 18 de setembro de 2019. Dispõe sobre as atribuições e competências do farmacêutico em serviços de hemoterapia e/ou bancos de sangue.,” 2019). These actions are essential to guarantee the effectiveness of the therapy, the reduction of safety issues, and the best utilization of the resources with minimum waste.

**Table 2:** Strategies to avoid emicizumab waste.

SUBJECT	DESCRIPTION
Compliance with recommendations and processes	Development or incorporation of a protocol to treat hemophilia A, including strategies to avoid product waste (D’Albini et al., 2023, Fasola et al., 2014, Srivastava et al., 2020, Yamada et al., 2020)
	Involvement of all the interdisciplinary team in the elaboration and implementation of best practices (D’Albini et al., 2023, Fasola et al., 2014, Srivastava et al., 2020, Yamada et al., 2020)
	Establishment of a management tool (e.g., Lean Six Sigma) to avoid drug waste (Damuzzo et al., 2019, Heitmiller et al., 2010)
	Close cooperation among assistant healthcare professionals (e.g., Physicians and Pharmacists) and decision makers to discuss the purchase and distribution of products to avoid waste (Berwick and Hackbarth, 2012)
Interdisciplinary team and treatment management	Continuing education for all the interdisciplinary team, discussing subjects such as treatment cost and drug waste (Srivastava et al., 2020, Tisdall et al., 2019, West and Cordina, 2019)
	Constant patient incentive for compliance with the proposed individualized therapy (Bekker et al., 2018, Bewley and Page, 2011, Srivastava et al., 2020)
Presentation and regimens	Availability of vials with different presentations (Hatswell and Porter, 2019)
	Sharing vials (Damuzzo et al., 2019, Ripoll Gallardo et al., 2015)
	Use of a device able to deliver the adequate dose (Asche et al., 2010; Bahannon et al., 1999; Wu et al., 2008)
	Evaluation of leftover expiry after reconstitution to make leftovers usable (Ripoll Gallardo et al., 2015, Romanelli and Lucente, 2022)
	Adaptation of the regimens to use pharmacokinetic-based and labeled-regimen proportional administration for complete vials (Donners et al., 2023, Liu et al., 2022)
	Adaptation of the regimens to use different doses for complete vials (Bansal et al., 2023, Chuansumrit et al., 2023)

Actions on product presentations, leftovers, and labeled regimens are the last alternative strategies. More vials with different amounts of emicizumab should be available (Hatswell and Porter, 2019). Vial sharing should be made possible at the healthcare institution (Damuzzo et al., 2019), guaranteeing the sterility of the product (Ripoll Gallardo et al., 2015). The stability of the leftovers after emicizumab reconstitution should be evaluated to provide the best expiry date and enable its reuse (Ripoll Gallardo et al., 2015, Romanelli and Lucente, 2022). Another option is the use of devices that deliver the adequate amount of the drug, such as pens and microneedle patches (Asche et al., 2010, Bohannon, 1999, Wu et al., 2008).

Finally, emicizumab regimens should be reassessed. In the Oncology setting, it is conceivable to round the dose to the closest entire vial. Experts state that dose rounds less or equal to 10 % of the prescribed dose are not expected to reduce the effectiveness or safety of therapy while reducing waste and costs (Fahrenbruch et al., 2018). However, the same experts highlight that additional studies to evaluate the impact of dose rounding on patient outcomes are warranted (Fahrenbruch et al., 2018). In the hemophilia setting, dose rounds to the nearest vials were suggested for PwHA with lower body weight (dose up and administration intervals increase) and higher body weight (dose down and administration intervals decrease) (Yu et al., 2021). Once more, the authors highlighted the need to evaluate the actual bleed prevention when adopting such a protocol (Yu et al., 2020). In parallel, since the pharmacokinetics of emicizumab allows regimens proportional to 1.5 mg per 1-kg of body weight per 7 days (Donners et al., 2021), different infusion frequencies have been used within 7 and 28 days (Donners et al., 2023, Liu et al., 2022). In this retrospective study, PwHA were treated with the label regimen and with the alternative pharmacokinetic-proportional regimen, to ensure the use of the entire vial (Donners et al., 2023). Annualized bleed rates reduced in comparison with the previous treatment based on factor infusion and were similar between the groups during emicizumab prophylaxis (Donners et al., 2023). A bleed rate reduction compared to the previous factor-based treatment was described in another cohort of PwHA who received a proportional regimen within 7 and 28 days ensuring the use of the entire vial (Liu et al., 2022). Nevertheless, the reduction of dose without pharmacokinetic proportionality but ensuring an entire vial use has also been described with a reduction of bleed rates compared to the previous factor-based treatment (Bansal et al., 2023, Chuansumrit et al., 2023).

### 4.1 Limitation

We did not have access to the real data PwHAi who failed ITI and were on emicizumab prophylaxis (Brasil, 2021). Consequently, we used alternative sources to build our model. However, we consider that these alternative sources were consistent, and our results can be a good approximation of the Brazilian reality.

## 5 Conclusion

In conclusion, the emicizumab prophylaxis for PwHAi who failed ITI in Brazil may generate waste as high as US$2,594,515, which represents 7.9 % of the reconstituted drug in the first year of treatment. Waste is more pronounced in the lower body weight and the shorter administration interval. Strategies to avoid waste would offer significant cost savings and must be urgently implemented given the continual pressure on resources within the Brazilian healthcare system.

## 6 Declaration of competing interests

RMC received speaker fees from Bayer, NovoNordisk, Hoffman-La Roche, and Takeda; consultancy fees from Hoffman-La Roche and Takeda, and scientific event grants from Bayer, NovoNordisk, Hoffman-La Roche, and Takeda. MMB, LCMH, APM, BG, AAGJ, FAA, and JAT declare no competing conflict of interest.

## CRediT authorship contribution statement

**Ricardo Mesquita Camelo:** Conceptualization, Data curation, Formal analysis, Investigation, Methodology, Resources, Visualization, Writing – original draft, Writing – review & editing. **Mariana Michel Barbosa:** Conceptualization, Data curation, Formal analysis, Investigation, Methodology, Resources, Visualization, Writing – original draft, Writing – review & editing. **Luila Clicia Moura Henriques:** . **Antony Paul Martin:** Visualization, Writing – review & editing. **Brian Godman:** Visualization, Writing – review & editing. **Augusto Afonso Guerra Júnior:** Visualization, Writing – review & editing. **Francisco de Assis Acurcio:** Visualization, Writing – review & editing. **Juliana Alvares-Teodoro:** Conceptualization, Data curation, Formal analysis, Investigation, Methodology, Resources, Visualization, Writing – original draft, Writing – review & editing.

## Declaration of competing interest

RMC received speaker fees from Bayer, NovoNordisk, Hoffman-La Roche, and Takeda; consultancy fees from Hoffman-La Roche and Takeda, and scientific event grants from Bayer, NovoNordisk, Hoffman-La Roche, and Takeda. MMB, LCMH, APM, BG, AAGJ, FAA, and JAT declare no competing conflict of interest.

## Footnotes

a Currency conversion according to the best exchange rate published on Dec/31/2019 (€1.00 = US$1.1239) at https://www.exchangerates.org.uk/EUR-USD-31_12_2019-exchange-rate-history.html. This date was arbitrarily chosen according to the date of the referenced article.
