# Supplementary material for: Emicizumab prophylaxis for people with hemophilia A: Waste estimation and the Brazilian perspective
Source: Saudi Pharm J. 2023 Nov 10;31(12):101867. doi: 10.1016/j.jsps.2023.101867 (PMC10661532; doi:10.1016/j.jsps.2023.101867)
Supplement: Supplementary data 1 [file mmc1.docx]

**Supplemental material**

**Emicizumab prophylaxis for people with hemophilia A: waste estimation and the Brazilian perspective**

Ricardo Mesquita Camelo, Mariana Michel Barbosa, Luila Clicia Moura Henriques, Antony Paul Martin, Brian Godman, Augusto Afonso Guerra Júnior, Francisco de Assis Acurcio, Juliana Alvares-Teodoro

DOI:XXX

Index

| **Legend** | **Excel spreadsheet** |
| --- | --- |
| **Supplemental Table sT1** Body weight percentiles according to the age range, by the World Health Organization. People older than 19 years were attributed to the body weight of a 19-year-old individual.  Available at <https://www.who.int/tools/child-growth-standards/standards/weight-for-age> (birth to 5 years) and <https://www.who.int/tools/growth-reference-data-for-5to19-years> (5 to 19 years)  P15, 15^th^ percentile body weight; P50, 50^th^ percentile body weight; P85, 85^th^ percentile body weight; WHO, World Health Organization. | sT1 |
| **Supplemental Table sT2** Maintenance regimen of emicizumab per body weight according to the CONITEC Protocol for Emicizumab Use (for the Brazilian Ministry of Health).  Available at <https://www.gov.br/saude/pt-br/assuntos/protocolos-clinicos-e-diretrizes-terapeuticas-pcdt/arquivos/2021/portal-portaria-conjuntano-15-pu-emicizumabe_hemofilia.pdf> (p.25)  MoH, Brazilian Ministry of Health; NA, not applicable; Q1W, every 1 week; Q2W, every 2 weeks. | sT2 |
| **Supplemental Table sT3** Results of the reconstituted, prescribed, and wasted emicizumab amounts (in mg) for loading and maintenance regimens during the first year of use per individual 1-kg body weight.  Negative values were considered 0 (zero) and reported as underdosage.  MoH, Brazilian Ministry of Health; NA, not applicable; Q1W, every 1 week; Q2W, every 2 weeks; Q4W, every 4 weeks. | sT3 |
| **Supplemental Table sT4** Results of the reconstituted, prescribed, and wasted emicizumab amounts and the percentage of wasted amount per reconstituted dose for loading and maintenance regimens during the first year of use per individual 1-kg body weight.  Negative values were considered 0 (zero) and reported as underdosage.  MoH, Brazilian Ministry of Health; NA, not applicable; Q1W, every 1 week; Q2W, every 2 weeks; Q4W, every 4 weeks. | sT4 |
| **Supplemental Table sT5** Estimation of the number of people with hemophilia A and inhibitors who failed immune tolerance induction, according to the BrazIT Study (availability of the age at immune tolerance induction failure and the number of individuals in each age range) and the CONITEC Recommendation Report for emicizumab (number of individuals who fulfilled the inclusion criteria for receiving emicizumab).  BrazIT, Brazilian Immune Tolerance; CONITEC, Comissão Nacional de Incorporação de Tecnologia (National Committee for Technology Incorporation); IT, immune tolerance induction. | sT5 |
| **Supplemental Table sT6** Costs of reconstituted and wasted emicizumab amounts for loading and maintenance regimens during the first year of use per age group based on body weight percentiles of the Brazilian people with hemophilia A and inhibitors who failed immune tolerance induction.  Negative values were considered 0 (zero) and reported as savings.  MoH, Brazilian Ministry of Health; P15, 15^th^ percentile body weight; P50, 50^th^ percentile body weight; P85, 85^th^ percentile body weight; Q1W, every 1 week; Q2W, every 2 weeks; Q4W, every 4 weeks; US$, United States dollar. | sT6 |
|  |  |
|  |  |
| **Legend** | **Word page** |
| **Supplemental Fig. s1** General information about emicizumab presentations, administration, and regimens. (**A**) Emicizumab presentations. (**B**) Emicizumab administration and regimens, according to the manufacturer recommendations and the CONITEC Protocol of Emicizumab Use for the Brazilian Ministry of Health.  CONITEC, Comissão Nacional de Incorporação de Tecnologia (National Committee for Technology Incorporation). | 3 |
| **Supplemental Fig. s2** Total wasted emicizumab after reconstitution per individual body weight, during the first year of treatment. The estimation included the loading doses and the maintenance regimens (every 1 week [Q1W], every 2 weeks [Q2W], every 4 weeks [Q4W], or hybrid), according to the recommendations and the choice of the vials proposed by the manufacturer and the Brazilian Ministry of Health. (**A**) Absolute wasted amount of emicizumab. (**B**) Percentage of wasted per reconstituted amount of emicizumab. | 4 |

**
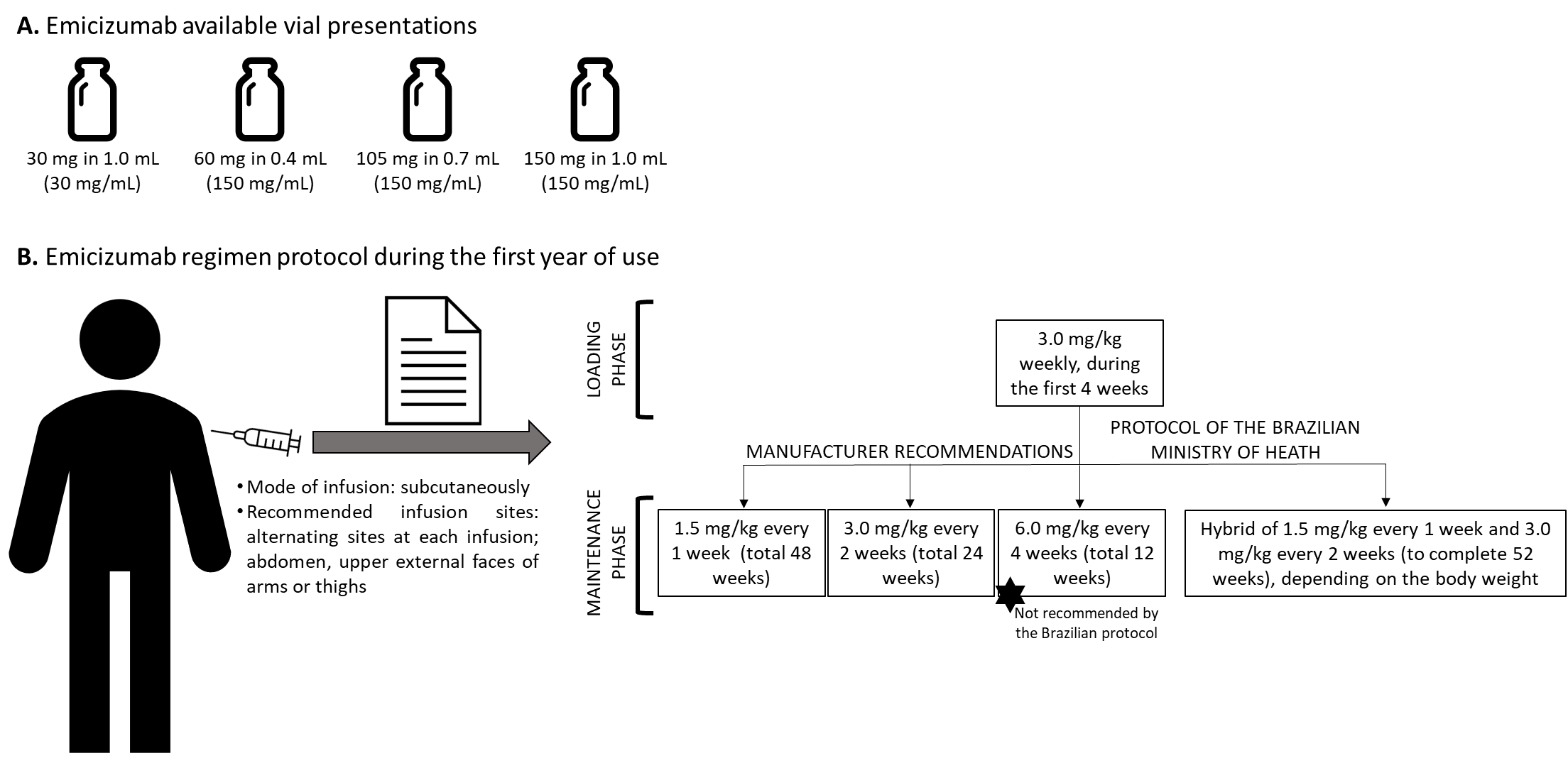
**

**Supplemental Fig. s1** General information about emicizumab presentations, administration, and regimens. (**A**) Emicizumab presentations. (**B**) Emicizumab administration and regimens, according to the manufacturer recommendations and the CONITEC Protocol of Emicizumab Use for the Brazilian Ministry of Health.

**
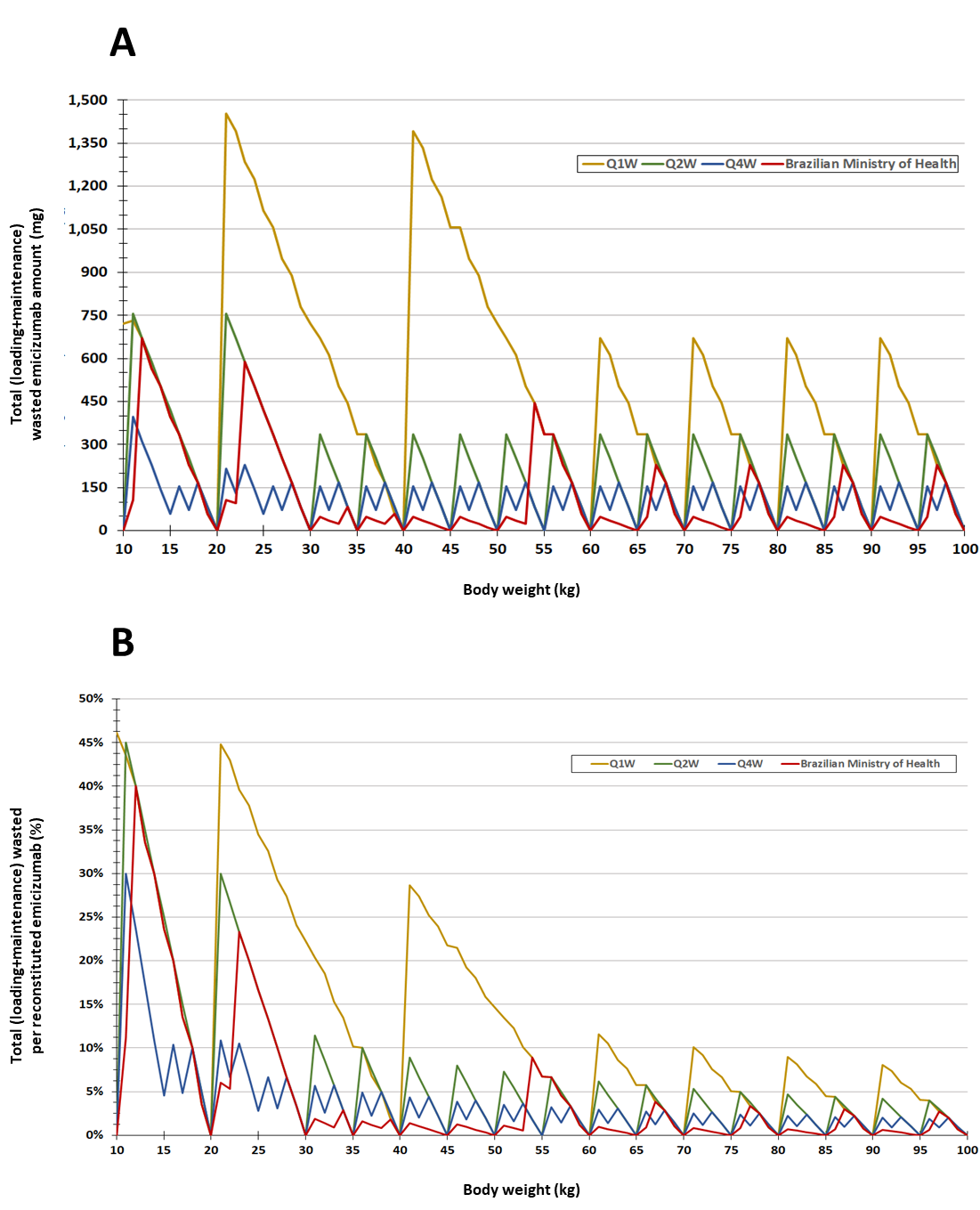
**

**Supplemental Fig. s2** Total wasted emicizumab after reconstitution per individual body weight, during the first year of treatment. The estimation included the loading doses and the maintenance regimens (every 1 week [Q1W], every 2 weeks [Q2W], every 4 weeks [Q4W], or hybrid), according to the recommendations and the choice of the vials proposed by the manufacturer and the Brazilian Ministry of Health. (**A**) Absolute wasted amount of emicizumab. (**B**) Percentage of wasted per reconstituted amount of emicizumab.
